# Resection of an Asymptomatic Lymphangioma in a 76-Year-Old Male

**DOI:** 10.7759/cureus.15577

**Published:** 2021-06-10

**Authors:** Paul E Creger, Charles Harper, Chelsea Curry, Adam Kramer

**Affiliations:** 1 Department of General Surgery, Kansas City University, Kansas City, USA; 2 Department of General Surgery, St. Mary's Medical Center, Blue Springs, USA; 3 Surgical Pathology, Centerpoint Medical Center, Independence, USA

**Keywords:** lymphangioma, mesentery, abdominal mass, lymphatics, small bowel

## Abstract

Lymphangiomas are benign congenital malformation comprised of the lymphatic system. They typically present in the head, neck, and axillary regions of children with <1% being described in the small bowel mesentery. We report a case of a 76-year-old man who presented with incidental large (9x6 cm) multiloculated cystic mass in the right upper quadrant (RUQ) on a CT scan performed for nephrolithiasis. He was asymptomatic at the presentation. We performed a diagnostic laparoscopy which was converted to an open procedure due to the mesenteric mass extending deeply toward the mesenteric root. The depth of invasion required small bowel resection with primary side-to-side anastomosis. Pathology confirmed a lymphangioma of the small bowel mesentery with histopathological analysis and cytology negative for malignant cells. Lymphangiomas are benign masses, however, their complete resection, including the resection of the involved organs is necessary. Incomplete resection or drainage is no longer used in management due to high rates of recurrence. Mesenteric lymphangiomas, while typically benign congenital malformations, can progress and impact surrounding structures via mass effect. Definitive treatment of lymphangiomas, even when asymptomatic, should be complete resection.

## Introduction

Lymphangiomas are uncommon, benign lymphatic malformations composed of thin-walled multiloculated cysts. They are primarily located in the head, neck, and axilla and are predominantly found in children [1]. Lymphangiomas occurring in the small bowel mesentery are extremely rare (<1%), especially in adults [2]. Mesenteric lymphangiomas are believed to arise from a congenital lymphatic malformation. Clinically they can present in a spectrum from completely asymptomatic up to similar symptomatology of acute abdomen [3].

## Case presentation

**Figure 1:**
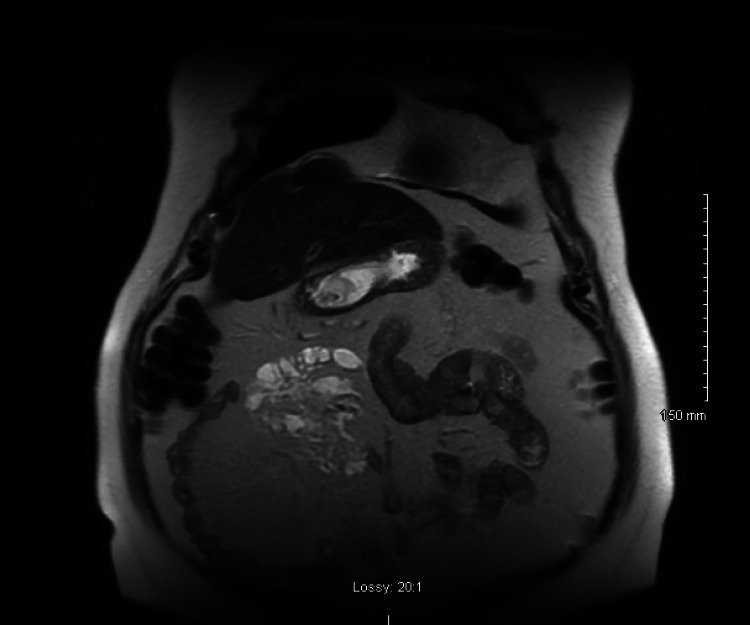
Coronal image of T2-weighted MRI of right upper quadrant lymphangioma with multiple loculations.

**Figure 2:**
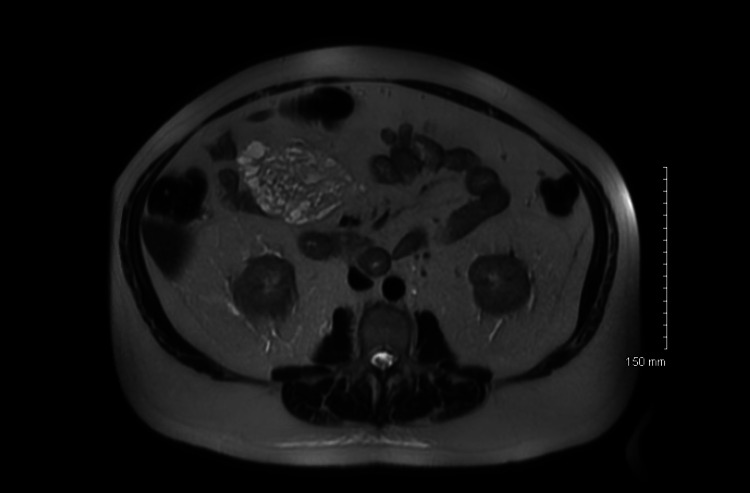
Axial image of T2-weighted MRI of right upper quadrant lymphangioma with multiple loculations.

A 76-year-old man was initially evaluated for a large kidney stone when CT noted an abnormal fluid-filled mass in the right upper quadrant (RUQ). Upon evaluation, he denied having any abdominal pain, early satiety, or changes in bowel habits. He had no other complaints at the time of the visit. He underwent an MRI which confirmed the CT findings and revealed a 9x6 cm multiloculated fluid-filled non-enhancing lesion typical of lymphangioma in the RUQ (Figures 1 and 2). The patient was informed of the risks and benefits of operative versus expectant management and decided to proceed with surgery.

The patient underwent diagnostic laparoscopy through a small infraumbilical Hasson entry. Initial inspection revealed multiple adhesions in the RUQ from the bowel and mesentery to the abdominal wall. Two more 5 mm trocars were placed in the left upper quadrant to assist in the lysis of adhesions using electrocautery and blunt dissection. The entire length of the small bowel was then inspected. A large, white appearing mass in the jejunal mesentery was encountered, consistent with the preoperative imaging. The mass was easily seen from both sides of the mesentery and appeared to extend downward to the mesenteric root. Due to the size and location of the mass, we decided to place a 5 cm supraumbilical laparoscopic hand assist port for easier palpation. Palpation of the mass determined it to be roughly 10 cm in diameter, irregular and soft in appearance and touch. The procedure was converted to open for better visualization of the mass. Elevating the mesenteric mass out of the abdomen allowed aspiration of fluid from the mass using an 18-gauge needle, which was sent for cytology. 

The degree of mesenteric involvement may have left the associated small bowel devascularized if resected alone, so a small bowel resection was performed. A stapled side-to-side anastomosis was performed. The mesenteric defect was closed. Fascia and skin were closed after final exploration and irrigation.

The patient had little postoperative pain. His postoperative course was uneventful, and he was discharged on postoperative day 2. At his two-week follow-up, the patient reported no complaints and was able to walk up to a mile daily.

**Figure 3:**
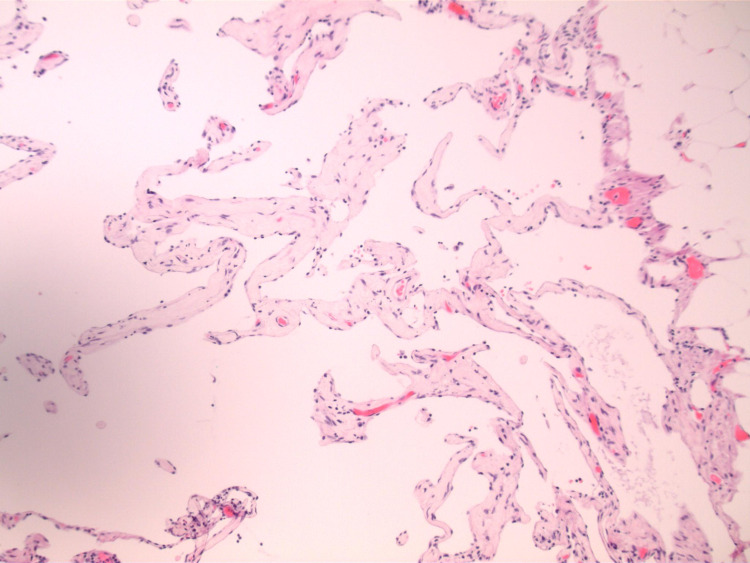
Focus on the lymphangioma within the small bowel mesentery.

Pathologic evaluation of the specimens revealed mesentery with a focus on lymphangioma (Figure 3). The small bowel had no significant pathologic abnormality and was negative for malignancy. The cytology specimen was also negative for malignancy.

## Discussion

Lymphangiomas are benign, lymphatic vessel malformations, primarily occurring in children in the head, neck, and axilla [1]. Other locations such as the abdomen or mediastinum are rare and account for roughly 5% of lymphangiomas, with small bowel mesenteric masses accounting for less than 1% [2,4]. Lymphangiomas are classically categorized into three types: capillary, cystic, and cavernous. Clinically, symptoms are non-specific and may range from asymptomatic to acute abdominal pain [3].

Differential diagnosis is broad, including lymphoma, lymphangiomyoma, secondary metastasis, and other rare mesenteric tumors like sarcomas and schwannomas [5]. Imaging studies like CT and ultrasound may be helpful in determining size and location, but histopathology is required to make a definitive diagnosis. Our patient had no history of trauma, previous abdominal surgery, or other possible predisposing factors and therefore required a diagnostic laparoscopy to obtain a definitive diagnosis.

While the patient described in this report was completely asymptomatic, the gold standard of treatment for lymphangiomas remains complete resection as they have the potential to enlarge to extreme sizes, invade surrounding structures, and have a malignant potential [5]. Should these masses expand deeper into the mesentery and surrounding small bowel, they can present with obstructive-like symptoms including abdominal distention, cramping, bloating, nausea, vomiting, and changes to bowel habitus [6]. Other management options such as drainage or partial resection, are no longer accepted treatment modalities as they carry a much higher recurrence rate that has been reported in around 10% [7].

## Conclusions

Lymphangiomas are rare in adult populations and even more rare in the small bowel mesentery. The patient presentation can vary greatly from completely asymptomatic to an acute abdomen. Complete resection is necessary due to the potential of expansion into adjacent structures, leading to obstruction or other complications. Definitive diagnosis following complete resection is confirmed with histopathology.
